# Current Awareness on Comparative and Functional Genomics

**DOI:** 10.1002/1097-0061(20000930)17:3<255::AID-YEA9>3.0.CO;2-7

**Published:** 2000


**Yeast**
*Yeast* 2000; **17:** 255–262

### Current awareness on comparative and functional genomics

### 1 Reviews & symposia

2000. Special issue: Database - 2000 Database issue. *Nucleic Acids Res* **28:** (1)

1999. Special issue: Repetitive DNA sequences in microbial genomes. *Res Microbiol* **150:** (9-10)

1999. Special section: Pharmacogenomics. *Mol Diagn* **4:** (4) 285.

Adams MD, Celniker SE, Holt RA, Evans CA, Gocayne JD, Amanatides PG, Scherer SE, Li PW, Hoskins RA, Galle RF *et al.* 2000. Celera Genomics, 45 West Gude Dr, Rockville, Md 20850, USA. The genome sequence of *Drosophila melanogaster* (Review). *Science* **287:** (5461) 2185.

Alizadeh AA, Staudt LM*. 2000. *NIH/NCI, Div Clin Sci, 9000 Rockville Pike, Bethesda, Md 20892, USA. Genomic-scale gene expression profiling of normal and malignant immune cells. *Curr Opin Immunol* **12:** (2) 219.

Attwood TK. 2000. Univ Manchester, Sch Biol Sci, Oxford Rd, Manchester M13 9PT, England. The quest to deduce protein function from sequence: The role of pattern databases (Review). *Int J Biochem Cell Biol* **32:** (2) 139.

Bakker BM, Westerhoff HV, Opperdoes FR, Michels PAM*. 2000. *Univ Catholique Louvain, Christian de Duve Inst Cellular Pathol, BE-1200 Brussels, Belgium. Metabolic control analysis of glycolysis in trypanosomes as an approach to improve selectivity and effectiveness of drugs (Mini-Review). *Mol Biochem Parasitol* **106:** (1) 1.

Bernardi G. 2000. Stn Zool, Lab Evoluz Mol, IT-80121 Naples, Italy. Isochores and the evolutionary genomics of vertebrates (Review). *Gene* **241:** (1) 3.

Bucher P. 1999. Swiss Inst Expt Canc Res, Ch Boveresses 166, CH-1066 Epalinges, Switzerland. Regulatory elements and expression profiles. *Curr Opin Struct Biol* **9:** (3) 400.

Carrilho E. 1999. Univ Sao Paulo, Inst Quim, Avda Dr Carlos Botelho 1465, CP 780, BR-13560-970 Sao Carlos, SP, Brazil. DNA sequencing by capillary array electrophoresis and microfabricated array systems. *Electrophoresis* **21:** (1) 55.

Copley RR, Schultz J, Ponting CP, Bork P. 1999. MBL, Meyerhofstr 1, DE-69012 Heidelberg, Germany. Protein families in multicellular organisms. *Curr Opin Struct Biol* **9:** (3) 408.

Danchin A. 1999. Inst Pasteur, 28 rue Dr Roux, FR-75724 Paris 15, France. From protein sequence to function. *Curr Opin Struct Biol* **9:** (3) 363.

Eisenstein E, Gilliland GL, Herzberg O, Moult J, Orban J, Poljak RJ, Banerjei L, Richardson D, Howard AJ. 2000. Univ Maryland, NIST, Inst Biotechnol, Ctr Adv Res Biotechnol, 9600 Gudelsky Dr, Rockville, Md 20850, USA. Biological function made crystal clear: Annotation of hypothetical proteins *via* structural genomics. *Curr Opin Biotechnol* **11:** (1) 25.

Epstein CB, Butow RA. 2000. Univ Texas, SW Med Ctr, Dept Mol Biol, 5323 Harry Hines Blvd, Dallas, Tx 75390, USA. Microarray technology: Enhanced versatility, persistent challenge. *Curr Opin Biotechnol* **11:** (1) 36.

Fickett JW, Wasserman WW. 2000. SmithKline Beecham Pharmaceut, Bioinformat Grp, 709 Swedeland Rd, King of Prussia, Pa 19406, USA. Discovery and modeling of transcriptional regulatory regions. *Curr Opin Biotechnol* **11:** (1) 19.

Fischer D, Eisenberg D. 1999. Fac Nat Sci, Dept Math & Comp Sci, IL-84015 Beer Sheva, Israel. Predicting structures for genome proteins. *Curr Opin Struct Biol* **9:** (2) 208.

Glynne RJ, Ghandour G, Goodnow CC. 2000. Eos Biotechnol, 225A Gateway Blvd, Sth San Francisco, Ca 94080, USA. Genomic-scale gene expression analysis of lymphocyte growth, tolerance and malignancy. *Curr Opin Immunol* **12:** (2) 210.

Griffin TJ, Smith LM. 2000. Univ Washington, Dept Mol Biotechnol, Box 357730, Seattle, Wa 98195, USA. Single-nucleotide polymorphism analysis by MALDI-TOF mass spectrometry (Review). *Trends Biotechnol* **18:** (2) 77.

Herschman HR, MacLaren DC, Iyer M, Namavari M, Bobinski K, Green LA, Wu L, Berk AJ, Toyokuni T, Barrio JR, Cherry SR, Phelps ME, Sandgren EP, Gambhir SS. 2000. UCLA, Mol Biol Inst 310, 611 Charles E Young Dr East, Los Angeles, Ca 90095, USA. Seeing is believing: Non-invasive, quantitative and repetitive imaging of reporter gene expression in living animals, using positron emission tomography (Mini-Review). *J Neurosci Res* **59:** (6) 699.

Hirabayashi T. 2000. Univ Tsukuba, Inst Biol Sci, Tennodai 1-1-1, Tsukuba, Ibaraki 305 8572, Japan. Agarose isoelectric focusing for the detection of many isoforms and high molecules in muscle protein analysis (Review). *Electrophoresis* **21:** (2) 446.

Jones DO, Cowell IG, Singh PB*. 2000. *Roslin Inst, Div Integrat Biol, Roslin EH25 9PS, Scotland. Mammalian chromodomain proteins: their role in genome organisation and expression (Review). *Bioessays* **22:** (2) 124.

Kelleher NL. 2000. Univ Illinois, Dept Chem, 600 Sth Mathews Ave, Urbana, Il 61801, USA. From primary structure to function: Biological insights from large-molecule mass spectra (Review). *Chem Biol* **7:** (2) R37.

Kidwell MG, Lisch DR. 2000. Univ Arizona, Dept Ecol & Evolutionary Biol, 116 BSW Bldg, Tucson, Az 85721, USA. Transposable elements and host genome evolution (Review). *Trends Ecol Evolut* **15:** (3) 95.

Ladunga I. 2000. SmithKline Beecham Pharmaceut, Bioinformat Dept, 709 Swedeland Rd, King of Prussia, Pa 19406, USA. Large-scale predictions of secretory proteins from mammalian genomic and EST sequences. *Curr Opin Biotechnol* **11:** (1) 13.

Lang BF, Gray MW, Burger G. 1999. Univ Montreal, Canadian Inst Adv Res, Dept Biochim, Prog Evolution Biol, CP 6128, Montreal, Quebec, Canada H3C 3J7. Mitochondrial genome evolution and the origin of eukaryotes. *Annu Rev Genet* **33:** 351.

Manabe T. 1999. Ehime Univ, Dept Chem, Matsuyama, Ehime 790 8577, Japan. Capillary electrophoresis of proteins for proteomic studies. *Electrophoresis* **20:** (15-16) 3116.

Manger ID, Relman DA*. 2000. *Stanford Univ, Dept Med, 299 Campus Dr, Stanford, Ca 94305, USA. How the host 'sees' pathogens: Global gene expression responses to infection. *Curr Opin Immunol* **12:** (2) 215.

Marrack P, Mitchell T, Hildeman D, Kedl R, Teague TK, Bender J, Rees W, Schaefer BC, Kappler J. 2000. Natl Jewish Med & Res Ctr, Dept Immunol, 1400 Jackson St, Denver, Co 80206, USA. Genomic-scale analysis of gene expression in resting and activated T-cells. *Curr Opin Immunol* **12:** (2) 206.

In order to keep subscribers up-to-date with the latest developments in their field, this current awareness service is provided by John Wiley & Sons and contains newly-published material on comparative and functional genomics. Each bibliography is divided into 16 sections. 1 Reviews & symposia; 2 General; 3 Large-scale sequencing and mapping; 4 Genome evolution; 5 Comparative genomics; 6 Gene families and regulons; 7 Pharmacogenomics; 8 Large-scale mutagenesis programmes; 9 Functional complementation; 10 Transcriptomics; 11 Proteomics; 12 Protein structural genomics; 13 Metabolomics; 14 Genomic approaches to development; 15 Technological advances; 16 Bioinformatics. Within each section, articles are listed in alphabetical order with respect to author. If, in the preceding period, no publications are located relevant to any one of these headings, that section will be omitted.

Copyright © 2000 John Wiley & Sons, Ltd.

Murzin AG, Patthy L. 1999. MRC Ctr Prot Engn, Hills Rd, Cambridge CB2 2QH, England. From sequence to structure to function: Editorial overview. *Curr Opin Struct Biol* **9:** (3) 359.

Myers EW, Sutton GG, Delcher AL, Dew IM, Fasulo DP, Flanigan MJ, Kravitz SA, Mobarry CM, Reinert KHJ, Remington KA *et al*. 2000. Celera Genomics Inc, 45 West Gude Dr, Rockville, Md 20850, USA. A whole-genome assembly of *Drosophila* (Review). *Science* **287:** (5461) 2196.

Norris SJ, Weinstock GM. 2000. Univ Texas, Dept Pathol & Lab Med, POB 20708, Houston, Tx 77225, USA. The genome sequence of *Treponema pallidum*, the syphilis spirochete: Will clinicians benefit?. *Curr Opin Infect Dis* **13:** (1) 29.

Orengo CA, Todd AE, Thornton JM. 1999. UCL, Biomol Struct & Modelling Unit, Gower St, London WC1E 6BT, England. From protein structure to function. *Curr Opin Struct Biol* **9:** (3) 374.

Ren J. 2000. Univ Bergen, Dept Pharmacol, Armauer Hansens Hus, NO-5021 Bergen, Norway. High-throughput screening of genetic mutations/polymorphisms by capillary electrophoresis (Review). *Comb Chem High Throughput Scr* **3:** (1) 11.

Rockett JC, Dix DJ. 2000. US/EPA, Reprod Toxicol Div, Res Triangle Pk, NC 27711, USA. DNA arrays: Technology, options and toxicological applications. *Xenobiotica* **30:** (2) 155.

Rosamond L, Allsop A*. 2000. *AstraZeneca, Alderley Pk, Macclesfield SK10 4TG, England. Harnessing the power of the genome in the search for new antibiotics (Review). *Science* **287:** (5460) 1973.

Rubin GM, Yandell MD, Wortman JR, Miklos GLG, Nelson CR, Hariharan IK, Fortini ME, Li PW, Apweiler R, Fleischmann W *et al*. 2000. Univ Calif, Lawrence Berkeley Lab, Drosophila Genome Project, Berkeley, Ca 94720, USA. Comparative genomics of the eukaryotes (Review). *Science* **287:** (5461) 2204.

Schmalzing S, Koutny L, Salas-Solano O, Adourian A, Matsudaira P, Ehrlich D. 1999. Whitehead Inst Biomed Res, Cambridge Ctr 9, Cambridge, Ma 02142, USA. Recent developments in DNA sequencing by capillary and microdevice electrophoresis. *Electrophoresis* **20:** (15-16) 3066.

Shapiro L, Harris T. 2000. CUNY Mount Sinai School Med, Struct Biology Program, 1425 Madison Ave, New York, NY 10029, USA. Finding function through structural genomics. *Curr Opin Biotechnol* **11:** (1) 31.

Sherlock G. 2000. Stanford Univ, Dept Genet, 300 Pasteur Dr, Stanford, Ca 94306, USA. Analysis of large-scale gene expression data. *Curr Opin Immunol* **12:** (2) 201.

Skolnick J, Fetrow JS, Kolinski A. 2000. Danforth Plant Sci Ctr, Lab Computat Genomics, 893 Nth Warson Rd, St Louis, Mo 63141, USA. Structural genomics and its importance for gene function analysis (Review). *Nat Biotechnol* **18:** (3) 283.

Sternberg MJE, Bates PA, Kelley LA, MacCallum RM. 1999. ICRF Biomolec Modelling Lab, 44 Lincolns Inn Fields, London WC2A 3PX, England. Prepress in protein structure prediction: Assessment of CASP3. *Curr Opin Struct Biol* **9:** (3) 368.

Stiles JK, Hicock PI, Shah PH, Meade JC*. 1999. *Univ Mississippi, Med Ctr, Dept Microbiol, 2500 Nth State St, Jackson, Ms 39216, USA. Genomic organization, transcription, splicing and gene regulation in *Leishmania* (Review). *Ann Trop Med Parasitol* **93:** (8) 781.

Sundberg SA. 2000. Caliper Technol Corp, 605 Fairchild Dr, Mt View, Ca 94043, USA. High-throughput and ultra-high-throughput screening: Solution- and cell-based approaches. *Curr Opin Biotechnol* **11:** (1) 47.

Tawata M, Aida K, Onaya T. 2000. Yamanashi Med Univ, Dept Internal Med III, Tamaho, Yamanashi 409 38, Japan. Screening for genetic mutations. A review. *Comb Chem High Throughput Scr* **3:** (1) 1.

Teichmann SA, Chothia C, Gerstein M. 1999. MRC Mol Biol Lab, Hills Rd, Cambridge CB2 2QH, England. Advances in structural genomics. *Curr Opin Struct Biol* **9:** (3) 390.

Templeton AR. 1999. Washington Univ, Dept Biol, CB 1137, St Louis, Mo 63130, USA. Uses of evolutionary theory in the human genome project. *Annu Rev Ecol Systematics* **30:** 23.

Weisberg RA, Gottesman ME, Hendrix RM, Little JW. 1999. NIH/NICHHD, Genet Mol Lab, Bethesda, Md 20892, USA. Family values in the age of genomics: Comparative analyses of temperate bacteriophage HK022. *Annu Rev Genet* **33:** 565.

### 2 General

Bihain BE, Shork N, Bougueleret L, Blumenfed M, Chumakov I, Yen F, Cohen D. 2000. Genset Corp, 857 Prospect St, La Jolla, Ca 92037, USA. Genomics: Promises and reality (French). *M S-Med Sci* **16:** (1) 17.

Cassier M. 2000. CERMES, 182 Blvd Villette, FR-75019 Paris, France. Relationships between public and private research in the area of genomics (French). *M S-Med Sci* **16:** (1) 26.

Jordan B. 2000. CNRS INSERM, Ctr Immunol, Case 906, FR-13288 Marseille 09, France. Genome, the ten-year itch (French). *M S-Med Sci* **16:** (1) 22.

### 3 Large-scale sequencing and mapping

Babinet C, Cohen-Tannoudji M. 2000. Inst Pasteur, CNRS URA 1960, Dev Biol Unit, 25 rue Dr Roux, FR-75015 Paris, France. Twenty years of programmed modifications of the mouse genome: A revolution in the genetic approach of mammalian biology (French). *M S-Med Sci* **16:** (1) 31.

Budiman MA, Mao L, Wood TC, Wing RA*. 2000. *Clemson Univ, Genomics Inst, Clemson, SC 29634, USA. A deep-coverage tomato BAC library and prospects toward development of an STC framework for genome sequencing. *Genome Res* **10:** (1) 129.

Cook JM, Martin J, Lewin A, Sinden RE, Tristem M. 2000. Univ London, Imperial Coll Sci & Technol, Dept Biol, Ascot SL5 7PY, England. Systematic screening of *Anopheles* mosquito genomes yields evidence for a major clade of *Pao*-like retrotransposons. *Insect Mol Biol* **9:** (1) 109.

D'Hont A, Paget-Goy A, Escoute J, Carreel F. 2000. CIRAD, BP 5035, FR-34032 Montpellier 1, France. The interspecific genome structure of cultivated banana, *Musa* spp. revealed by genomic DNA *in situ* hybridization. *Theor Appl Genet* **100:** (2) 177.

Fazeli CF, Rezaian MA*. 2000. *CSIRO, Plant Ind & Cooperat Res Ctr Viticult, POB 350, Glen Osmond, SA 5064, Australia. Nucleotide sequence and organization of ten open reading frames in the genome of grapevine leafroll-associated virus 1 and identification of three subgenomic RNAs. *J Gen Virol* **81:** (3) 605.

Gostimsky SA, Kokaeva ZG, Bobrova VK. 1999. MV Lomonosov State Univ, Dept Genet & Breeding, RU-119899 Moscow, Russia. Use of molecular markers for the analysis of plant genome. *Russ J Genet* **35:** (11) 1326.

Griffin DK, Haberman F, Masabanda J, O'Brien P, Bagga M, Sazanov A, Smith J, Burt DW, Ferguson-Smith M, Wienberg J. 1999. Brunel Univ, Dept Biol Sci, Uxbridge UB8 3PH, England. Micro- and macrochromosome paints generated by flow cytometry and microdissection: Tools for mapping the chicken genome. *Cytogenet Cell Genet* **87:** (3-4) 278.

Groenen MAM, Cheng HH, Bumstead N, Benkel BF, Briles WE, Burke T, Burt DW, Crittenden LB, Dodgson J, Hillel J, Lamont S, De Leon AP, Soller M, Takahashi H, Vignal A. 2000. Agr Univ, Inst Anim Sci, Anim Breeding & Genet Grp, NL-6709 PG Wageningen, The Netherlands. A consensus linkage map of the chicken genome. *Genome Res* **10:** (1) 137.

Hashimoto Y, Hayashi K, Hayakawa T, Ueno Y, Shimojo E, Kondo A, Miyasono M, Sano Y, Matsumoto T, Granados RR. 2000. Kyoto Inst Technol, Dept Appl Biol, Kyoto 606 8585, Japan. Physical map of a *Plutella xylostella* granulovirus genome. *Appl Entomol Zool* **35:** (1) 45.

Kokotovic B, Bolske G, Ahrens P, Johansson KE. 2000. Danish Vet Lab, Bullowsvej 27, DK-1790 Copenhagen V, Denmark. Genomic variations of *Mycoplasma capricolum* subsp *capripneumoniae* detected

Copyright © 2000 John Wiley & Sons, Ltd.

by amplified fragment length polymorphism (AFLP) analysis. *FEMS Microbiol Lett* **184:** (1) 63.

Kornberg TB, Krasnow M. 2000. Univ Calif, Dept Biochem & Biophys, San Francisco, Ca 94143, USA. The Drosophila genome sequence: Implications for biology and medicine. *Science* **287:** (5461) 2218.

Manabe YC, Dannenberg AM, Bishai WR*. 2000. *Johns Hopkins Univ, Ctr TB Res, 615 Nth Wolfe St, Baltimore, Md 21205, USA. What we can learn from the *Mycobacterium tuberculosis* genome sequencing projects. *Int J Tuberc Lung Dis* **4:** (2 Suppl 1) S18.

Osoegawa K, Tateno M, Woon PY, Frengen E, Mammoser AG, Catanese JJ, Hayashizaki Y, De Jong PJ*. 2000. *Roswell Pk Canc Inst, Dept Canc Genet, Buffalo, NY 14263, USA. Bacterial artificial chromosome libraries for mouse sequencing and functional analysis. *Genome Res* **10:** (1) 116.

Parkhill J, Wren BW, Mungall K, Ketley JM, Churcher C, Basham D, Chillingworth T, Davies RM, Feltwell T, Holroyd S, Jagels K, Kardyshev AV, Moule S, Pallen MJ, Penn CW, Quail MA, Rajandream MA, Rutherford KM, Van Vliet AHM, Whitehead S, Barrell BG. 2000. Sanger Centre, Wellcome Trust Genome Campus, Cambridge CB10 1SA, England. The genome sequence of the food-borne pathogen *Campylobacter jejuni* reveals hypervariable sequences. *Nature* **403:** (6770) 665.

Potekhin AA, Rautian MS, Brigge T. 1999. St Petersburg State Univ, Biol Res Inst, Staryi Peterhof, RU-198904 St Petersburg, Russia. The genome of *Paramecium caudatum*: Analysis by means of pulsed field gel electrophoresis. *Russ J Genet* **35:** (12) 1413.

Raghavan S, Hariharan R, Brahmachari SK*. 2000. *Ctr Biochem Technol, Funct Genomics Unit, Mall Rd, IN-110007 Delhi, India. Polypurine-polypyrimidine sequences in complete bacterial genomes: Preference for polypurines in protein-coding regions. *Gene* **242:** (1-2) 275.

Rubin GM, Lewis EB. 2000. Univ Calif, Lawrence Berkeley Lab, Drosophila Genome Project, Berkeley, Ca 94720, USA. A brief history of *Drosophila's* contributions to genome research. *Science* **287:** (5461) 2216.

Tettelin H, Saunders NJ, Heidelberg J, Jeffries AC, Nelson KE, Eisen JA, Ketchum KA, Hood DW, Peden JF, Dodson RJ, et al. 2000. Inst Genom Res, 9712 Med Ctr Dr, Rockville, Md 20850, USA. Complete genome sequence of *Neisseria meningitidis* serogroup B strain MC58. *Science* **287:** (5459) 1809.

Van Helden J, Del Olmo M, Perez-Ortin JE. 2000. Free Univ Brussels, Unite Conformat Macromol Biol, CP 160-16, 50 Ave FD Roosevelt, BE-1050 Brussels, Belgium. Statistical analysis of yeast genomic downstream sequences reveals putative polyadenylation signals. *Nucleic Acids Res* **28:** (4) 1000.

Weissenbach J, Salanoubat M. 2000. Genoscope, 2 rue Gaston Cremieux, FR-91057 Evry, France. Genome sequencing: The big bang (French). *M S-Med Sci* **16:** (1) 10.

### 4 Genome evolution

Chetelat RT, Meglic V, Cisneros P. 2000. Univ Calif Davis, Dept Vegetable Crops, 1 Shields Ave, Davis, Ca 95616, USA. A genetic map of tomato based on BC1 *Lycopersicon esculentum* x *Solanum lycopersicoides* reveals overall synteny but suppressed recombination between these homeologous genomes. *Genetics* **154:** (2) 857.

Gibson TJ, Spring J. 2000. EMBL, Postfach 102209, DE-69012 Heidelberg, Germany. Evidence in favour on ancient octaploidy in the vertebrate genome. *Biochem Soc Trans* **28:** (2) 259.

Hughes DC. 2000. Birmingham Women's Hosp, Assisted Concept Unit, Reprod Biol & Genet Grp, Birmingham B15 2TG, England. MIRs as agents of mammalian gene evolutions. *Trends Genet* **16:** (2) 60.

Katz LA, Curtis EA, Pfunder M, Landweber LF. 2000. Smith Coll, Dept Sci Biol, Northampton, Ma 01063, USA. Characterization of novel sequences from distantly related taxa by walking PCR. *Mol Phylogenet Evol* **14:** (2) 318.

Lang BF, Paquin B, Burger G. 2000. Univ Montreal, Inst Canadian Rech Adv, Dept Biochim, Prog Biol Evolut, Montreal, Quebec, Canada H3T 1J4. Molecular evolution and the genomics revolution (French, English Abstract). *M S-Med Sci* **16:** (2) 212.

Lozovskaya ER, Nurminsky D, Petrov DA, Hartl DL*. 1999. *Harvard Univ, Dept Organism & Evolutionary Biol, Cambridge, Ma 02138, USA. Genome size as a mutation-selection-drift process. *Genes Genet Syst* **74:** (5) 201.

Marmiroli N, Maestri E, Liviero L, Massari A, Malcevschi A, Moniciardini P. 1999. Univ Parma, Div Genet & Environm Biotechnol, IT-43100 Parma, Italy. Application of genomics in assessing biodiversity in wild and cultivated barley. *Mol Ecol* **8:** (12 Suppl 1) S95.

Robertson HM. 2000. Univ Illinois, Dept Entomol, 320 Morrill Hall, Urbana, Il 61801, USA. The large srh family of chemoreceptor genes in *Caenorhabditis* nematodes reveals processes of genome evolution involving large duplications and deletions and intron gains and losses. *Genome Res* **10:** (2) 192.

Salemi M, Desmyter J, Vandamme AM. 2000. Catholic Univ, Rega Inst Med Res, Minderbroedersstr 10, BE-3000 Louvain, Belgium. Tempo and mode of human and simian T-lymphotropic virus (HTLV/STLV) evolution revealed by analyses of full-genome sequences. *Mol Biol Evol* **17:** (3) 374.

Tosi LRO, Beverley SM*. 2000. *Washington Univ, Dept Mol Microbiol, 660 Sth Euclid Ave, St Louis, Mo 63110, USA. *Cis* and *trans* factors affecting *Mos1 mariner* evolution and transposition *in vitro*, and its potential for functional genomics. *Nucleic Acids Res* **28:** (3) 784.

### 5 Comparative genomics

Cao Y, Kim KS, Ha JH, Hasegawa M. 1999. Inst Stat Math, Minato ku, 4-6-7 Minami Azabu, Tokyo 106 8569, Japan. Model dependence of the phylogenetic influence: Relationship among Carnivores, Perissodactyls and Cetartiodactyls as inferred from mitochondrial genome sequences. *Genes Genet Syst* **74:** (5) 211.

Devos KM, Pittaway TS, Reynolds A, Gale MD. 2000. John Innes Ctr Plant Sci Res, Norwich Res Pk, Norwich NR4 7UH, England. Comparative mapping reveals a complex relationship between the pearl millet genome and those of foxtail millet and rice. *Theor Appl Genet* **100:** (2) 190.

Perriere G, Duret L, Gouy M. 2000. Univ Lyon 1, Lab Biometrie & Biol Evolut, CNRS UMR 5558, FR-69622 Villeurbanne, France. HOBACGEN: Database system for comparative genomics in bacteria. *Genome Res* **10:** (3) 379.

Read TD, Brunham RC, Shen C, Gill SR, Heidelberg JF, White O, Hickey EK, Peterson J, Utterback T, Berry K, Bass S, Linher K, Weidman J, Khouri H, Craven B, Bowman C, Dodson R, Gwinn M, Nelson W, DeBoy R, Kolonay J, McClarty G, Salzberg SL, Eisen J, Fraser CM*. 2000. *Inst Genomic Res, 9712 Med Ctr Dr, Rockville, Md 20850, USA. Genome sequences of *Chlamydia trachomatis* MoPn and *Chlamydia pneumoniae* AR39. *Nucleic Acids Res* **28:** (6) 1397.

Rohwer F, Segall A, Steward G, Seguritan V, Breitbart M, Wolven F, Azam F. 2000. Scripps Inst Oceanog, Div Marine Biol Res, La Jolla, Ca 92037, USA. The complete genomic sequence of the marine phage Roseophage SIO1 shares homology with nonmarine phages. *Limnol Oceanogr* **45:** (2) 408.

Schoen DJ. 2000. McGill Univ, Dept Biol, 1205 Dr Penfield Ave, Montreal, Quebec, Canada H3A 1B1. Comparative genomics, marker density and statistical analysis of chromosome rearrangements. *Genetics* **154:** (2) 943.

Subramanian G, Koonin EV*, Aravind L. 2000. *NIH, Natl Ctr Biotechnol Informat, Bethesda, Md 20894, USA. Comparative genome analysis of the pathogenic spirochetes *Borrelia burgdorferi* and *Treponema pallidum*. *Infect Immun* **68:** (3) 1633.

Vilei EM, Abdo EM, Nicolet J, Botelho A, Goncalves R, Frey J*. 2000. *Univ Bern, Inst Vet Bacteriol, Langgass Str 122, CH-3012 Bern, Switzerland. Genomic and antigenic differences between the European

Copyright © 2000 John Wiley & Sons, Ltd.

and African/Australian clusters of *Mycoplasma mycoides* subsp *mycoides* SC. *Microbiology* **146:** (2) 477.

Yang F, O'Brien PCM, Milne BS, Graphodatsky AS, Solanky N, Trifonov V, Rens W, Sargan D, Ferguson-Smith MA*. 1999. *Univ Cambridge, Dept Clin Vet Med, Madingley Rd, Cambridge CB3 0ES, England. A complete comparative chromosome map for the dog, red fox, and human and its integration with canine genetic maps. *Genomics* **62:** (2) 189.

Zabarovska V, Li JF, Muravenko O, Fedorova L, Braga E, Ernberg I, Wahlstedt C, Klein G, Zabarovsky ER*. 2000. *Karolinska Inst, Ctr Microbiol & Tumor Biol, Box 280, SE-17177 Stockholm, Sweden. CIS: Cloning of identical sequences between two complex genomes. *Chromosome Res* **8:** (1) 77.

### 6 Gene families and regulons

Alba R, Kelmenson PM, Cordonnier-Pratt MM*, Pratt LH. 2000. *Univ Georgia, Dept Bot, Athens, Ga 30602, USA. The phytochrome gene family in tomato and the rapid differential evolution of this family in angiosperms. *Mol Biol Evol* **17:** (3) 362.

Conticello SG, Pilpel Y, Glusman G, Fainzilber M. 2000. Weizmann Inst Sci, Dept Biol Chem, Mol Neurobiol Lab, IL-76100 Rehovot, Israel. Position-specific codon conservation in hypervariable gene families. *Trends Genet* **16:** (2) 57.

Goerke C, Campana S, Bayer MG, Doring G, Botzenhart K, Wolz C*. 2000. *Univ Tubingen Allgemeine Hyg & Umwelthyg, Wilhelmstr 31, DE-72074 Tubingen, Germany. Direct quantitative transcript analysis of the *agr* regulon of *Staphylococcus aureus* during human infection in comparison to the expression profile *in vitro*. *Infect Immun* **68:** (3) 1304.

Kruglyak S, Tang HX. 2000. Univ Sthn Calif, Dept Math, Los Angeles, Ca 90089, USA. Regulation of adjacent yeast genes. *Trends Genet* **16:** (3) 109.

Sankoff D. 1999. Univ Montreal, Ctr Rech Math, Succursale Ctr-Ville, CP 6128, Montreal, Quebec, Canada H3C 3J7. Genome rearrangement with gene families. *Bioinformatics* **15:** (11) 909.

Sugino H, Hamada S, Yasuda R, Tuji A, Matsuda Y, Fujita M, Yagi T*. 2000. *Natl Inst Physiol Sci, Lab Neurobiol & Behav Genet, Okazaki, Aichi 444 8585, Japan. Genomic organization of the family of *CNR* cadherin genes in mice and humans. *Genomics* **63:** (1) 75.

Vanhanen S, West M, Kroon JTM, Lindner N, Casey J, Cheng Q, Elborough KM, Slabas AR*. 2000. *Univ Durham, Dept Biol Sci, South Rd, Durham DH1 3LE, England. A consensus sequence for long-chain fatty-acid alcohol oxidases from *Candida* identifies a family of genes involved in lipid ω-oxidation in yeast with homologues in plants and bacteria. *J Biol Chem* **275:** (6) 4445.

### 7 Pharmacogenomics

Germer S, Holland MJ, Higuchi R. 2000. Roche Mol Syst, Alameda, Ca 94501, USA. High-throughput SNP allele-frequency determination in pooled DNA samples by kinetic PCR. *Genome Res* **10:** (2) 258.

Islam MQ, Islam K. 2000. Linkoping Univ, Dept Biomed & Genet, SE-58185 Linkoping, Sweden. A new functional classification of tumor-suppressing genes and its therapeutic implications. *Bioessays* **22:** (3) 274.

Loferer H, Jacobi A, Posch A, Gauss C, Meier-Ewert S, Seizinger B. 2000. Genome Pharmaceut Corp, Fraunhoferstr 20, DE-82152 Martinsried, Germany. Integrated bacterial genomics for the discovery of novel antimicrobials. *Drug Discov Today* **5:** (3) 107.

Mein CA, Barratt BJ, Dunn MG, Siegmund T, Smith AN, Esposito L, Nutland S, Stevens HE, Wilson AJ, Phillips MS, Jarvis N, Law S, De Arruda M*, Todd JA. 2000. *Univ Cambridge, Addenbrookes Hosp, Wellcome Trust Ctr Study Mol Mech Dis, Cambridge CB2 2XY, England. Evaluation of single nucleotide polymorphism typing with Invader on PCR amplicons and its automation. *Genome Res* **10:** (3) 330.

Nessling M, Kern MA, Schadendorf D, Lichter P*. 1999. *Deutsch Krebsforschungszentrum, Abt Org Komplex Genome, Neuenheimer Feld 280, DE-69120 Heidelberg, Germany. Association of genomic imbalances with resistance to therapeutic drugs in human melanoma cell lines. *Cytogenet Cell Genet* **87:** (3-4) 286.

Pizza M, Scarlato V, Masignani V, Giuliani MM, Arico B, Comanducci M, Jennings GT, Baldi L, Bartolini E, Capecchi B, et al. 2000. c/o Rappuoli R, Chiron SpA, Immunobiol Res Inst, via Fiorentina 1, IT-53100 Siena, Italy. Identification of vaccine candidates against serogroup B meningococcus by whole-genome sequencing. *Science* **287:** (5459) 1816.

Srivastava M, Eidelman O, Pollard HB*. 1999. *Uniformed Serv Univ Hlth Sci, Dept Anat & Cell Biol, 4301 Jones Bridge Rd, Bethesda, Md 20814, USA. Pharmacogenomics of the cystic fibrosis transmembrane conductance regulator (CFTR) and the cystic fibrosis drug CPX using genome microarray analysis. *Mol Med* **5:** (11) 753.

### 8 Large-scale mutagenesis programmes

Chen YJ, Yee D, Dains K, Chatterjee A, Cavalcoli J, Schneider E, Om J, Woychik RP, Magnuson T*. 2000. *Case Western Reserve Univ, Dept Genet, Cleveland, Oh 44106, USA. Genotype-based screen for ENU-induced mutations in mouse embryonic stem cells. *Nat Genet* **24:** (3) 314.

### 10 Transcriptomics

Bortoluzzi S, D'Alessi F, Romualdi C, Danieli GA*. 2000. *Univ Padua, Dept Biol, IT-35131 Padua, Italy. The human adult skeletal muscle transcriptional profile reconstructed by a novel computational approach. *Genome Res* **10:** (3) 344.

Clayton C. 2000. Univ Heidelberg, Zentrum Mol Biol, Neuenheimer Feld 282, DE-69120 Heidelberg, Germany. Why do we need standard genetic nomenclature for parasite genes and gene products? *Acta Trop* **75:** (1) 119.

Geiss GK, Bumgarner RE, An MC, Agy MB, Van't Wout AB, Hammersmark E, Carter VS, Upchurch D, Mullins JI, Katze MG*. 2000. *Univ Washington, Dept Microbiol, Box 357330, Seattle, Wa 98195, USA. Large-scale monitoring of host cell gene expression during HIV-1 infection using cDNA microarrays. *Virology* **266:** (1) 8.

Gelfand MS, Koonin EV, Mironov AA. 2000. State Sci Ctr Biotechnol NIIGenetika, RU-113545 Moscow, Russia. Prediction of transcription regulatory sites in Archaea by a comparative genomic approach. *Nucleic Acids Res* **28:** (3) 695.

Hasty J, Pradines J, Dolnik M, Collins JJ. 2000. Boston Univ, Ctr Biodynam, 44 Cummington St, Boston, Ma 02215, USA. Noise-based switches and amplifiers for gene expression. *Proc Natl Acad Sci U S A* **97:** (5) 2075.

Henikoff S. 2000. Howard Hughes Med Inst, Fred Hutchinson Canc Res Ctr, Seattle, Wa 98109, USA. Heterochromatin function in complex genomes. *Biochim Biophys Acta* **1470:** (1) O1.

Hipfel R, Schittek B, Bodingbauer Y, Garbe C*. 2000. *Univ Tubingen, Sect Dermatol Oncol, Liebermeisterstr 25, DE-72076 Tubingen, Germany. Specifically regulated genes in malignant melanoma tissues identified by subtractive hybridization. *Br J Cancer* **82:** (6) 1149.

Jansen R, Gerstein M*. 2000. *Yale Univ, Dept Mol Biophys & Biochem, 266 Whitney Ave, POB 208114, New Haven, Ct 06520, USA. Analysis of the yeast transcriptome with structural and functional categories: Characterizing highly expressed proteins. *Nucleic Acids Res* **28:** (6) 1481.

Kuno N, Muramatsu T, Hamazato F, Furuya M*. 2000. *Hitachi Adv Res Lab, Hatoyama, Saitama 350 0395, Japan. Identification by large-scale screening of phytochrome-regulated genes in etiolated seedlings of *Arabidopsis* using a fluorescent differential display technique. *Plant Physiol* **122:** (1) 15.

Copyright © 2000 John Wiley & Sons, Ltd. 

Larsson M, Stahl S, Uhlen M, Wennborg A. 2000. Royal Inst Technol, Dept Biotechnol, Teknikringen 34, SE-10044 Stockholm, Sweden. Expression profile viewer (ExProView): A software tool for transcriptome analysis. *Genomics* **63:** (3) 341.

Louie AY, Huber MM, Ahrens ET, Rothbacher U, Moats R, Jacobs RE, Fraser SE, Meade TJ*. 2000. *Calif Inst Technol, Beckman Inst, Div Biol, Pasadena, Ca 91125, USA. *In vivo* visualization of gene expression using magnetic resonance imaging. *Nat Biotechnol* **18:** (3) 321.

Morrison B, Eberwine JH, Meaney DF, McIntosh TK*. 2000. *3320 Smith Walk, Hayden Hall, Room 105C, Philadelphia, Pa 19104, USA. Traumatic injury induces differential expression of cell death genes in organotypic brain slice cultures determined by complementary DNA array hybridization. *Neuroscience* **96:** (1) 131.

Ross DT, Scherf U, Eisen MB, Perou CM, Rees C, Spellman P, Iyer V, Jeffrey SS, Van de Rijn M, Waltham M, Pergamenschikov A, Lee JCE, Lashkari D, Shalon D, Myers TG, Weinstein JN, Botstein D, Brown PO*. 2000. *Stanford Univ, Dept Biochem, Stanford, Ca 94305, USA. Systematic variation in gene expression patterns in human cancer cell lines. *Nat Genet* **24:** (3) 227.

Scherf U, Ross DT, Waltham M, Smith LH, Lee JK, Tanabe L, Kohn KW, Reinhold WC, Myers TG, Andrews DT, Scudiero DA, Eisen MB, Sausville EA, Pommier Y, Botstein D, Brown PO, Weinstein JN*. 2000. *NIH/NCI, Mol Pharmacol Lab, 9000 Rockville Pike, Bethesda, Md 20892, USA. A gene expression database for the molecular pharmacology of cancer. *Nat Genet* **24:** (3) 236.

Villaret DB, Wang TT, Dillon D, Xu JC, Sivam D, Cheever MA, Reed SG. 2000. Univ Florida, Dept Otolaryngol, 1600 SW Archer Rd, Gainesville, Fl 32610, USA. Identification of genes overexpressed in head and neck squamous cell carcinoma using a combination of complementary DNA subtraction and microarray analysis. *Laryngoscope* **110:** (3 Pt 1) 374.

Voehringer DW, Hirschberg DL, Xiao J, Lu Q, Roederer M, Lock CB, Herzenberg LA, Steinman L, Herzenberg LA. 2000. Stanford Univ, Dept Genet, Beckman Ctr, Stanford, Ca 94305, USA. Gene microarray identification of redox and mitochondrial elements that control resistance or sensitivity to apoptosis. *Proc Natl Acad Sci U S A* **97:** (6) 2680.

Zanders ED, Goulden MG, Kennedy TC, Kempsell KE. 2000. Glaxo Wellcome R&D Ltd, Immunopathol Unit, Gunnels Wood Rd, Stevenage SG1 2NY, England. Analysis of immune system gene expression in small rheumatoid arthritis biopsies using a combination of subtractive hybridization and high-density cDNA arrays. *J Immunol Methods* **233:** (1-2) 131.

### 11 Proteomics

Bergman AC, Benjamin T, Alaiya A, Waltham M, Sakaguchi K, Franzen B, Linder S, Bergman T, Auer G, Appella E, Wirth PJ, Jornvall H*. 2000. Karolinska Inst, Dept Med Biochem & Biophys, SE-17177 Stockholm, Sweden. Identification of gel-separated tumor marker proteins by mass spectrometry. *Electrophoresis* **21:** (3) 679.

Bhasin VK. 2000. Univ Delhi, Dept Zool, IN-110007 Delhi, India. Proteomics could be key in battle against malaria (Letter). *Nature* **403:** (6771) 698.

Boussac M, Garin J. 2000. CEA, Dept Biol Mol & Struct, 17 rue Martyrs, FR-38054 Grenoble 9, France. Calcium-dependent secretion in human neutrophils: A proteomic approach. *Electrophoresis* **21:** (3) 665.

Colas P. 2000. Ecole Normale Super Lyon, Lab Biol Cellulaire & Mol, 46 Allee Italie, FR-69364 Lyon 07, France. Genome-scale exploration of protein interactions (French). *M S-Med Sci* **16:** (1) 50.

Cutler P, Bell DJ, Birrell HC, Connelly JC, Connor SC, Holmes E, Mitchell BC, Monte SY, Neville BA, Pickford R, Polley S, Schneider K, Skehel JM. 1999. SmithKline Beecham Pharmaceut, Dept Analyt Sci, Harlow CM19 5AW, England. An integrated proteomic approach to studying glomerular nephrotoxicity. *Electrophoresis* **20:** (18) 3647.

Dainese-Hatt P, Fischer HM, Hennecke H, James P*. 1999. *ETH Zentrum, Prot Chem Lab, Universitatsstr 16, CH-8092 Zurich, Switzerland. Classifying symbiotic proteins from *Bradyrhizobium japonicum* into functional groups by proteome analysis of altered gene expression levels. *Electrophoresis* **20:** (18) 3514.

Dunn MJ. 2000. Univ London, Imperial Coll Sci Technol & Med, Harefield Hosp, Heart Sci Ctr, Harefield UB9 6JH, England. Studying heart disease using the proteomic approach. *Drug Discov Today* **5:** (2) 76.

Flajolet M, Rotondo G, Daviet L, Bergametti F, Inchauspe G, Tiollais P, Transy C, Legrain P*. 2000. *Inst Pasteur, Unite Genet Interact Macromol, CNRS URA 1300, 25 rue Dr Roux, FR-75724 Paris 15, France. A genomic approach of the hepatitis C virus generates a protein interaction map. *Gene* **242:** (1-2) 369.

Hayakawa T, Kojima K, Nonaka K, Nakagaki M, Sahara K, Asano S, Iizuka T, Bando H*. 2000. *Hokkaido Univ, Div Appl Biosci, Sapporo, Hokkaido 060, Japan. Analysis of proteins encoded in the bipartite genome of a new type of parvo-like virus isolated from silkworm: Structural protein with DNA polymerase motif. *Virus Res* **66:** (1) 101.

Ito T, Tashiro K, Muta S, Ozawa R, Chiba T, Nishizawa M, Yamamoto K, Kuhara S, Sakaki Y. 2000. Univ Tokyo, Ctr Human Genome, Minato ku, 4-6-1 Shirokanedai, Tokyo 108 8639, Japan. Toward a protein-protein interaction map of the budding yeast: A comprehensive system to examine two hybrid interactions in all possible combinations between the yeast proteins. *Proc Natl Acad Sci U S A* **97:** (3) 1143.

Kabani M, Boisrame A, Beckerich JM, Gaillardin C. 2000. INRA, INAPG, CNRS, Lab Genet Mol & Cellulaire, BP 01, FR-78850 Thiverval Grignon, France. A highly representative two-hybrid genomic library for the yeast *Yarrowia lipolytica*. *Gene* **241:** (2) 309.

Kanitz MH, Witzmann FA, Zhu H, Fultz CD, Skaggs S, Moorman WJ, Savage RE. 1999. NIOSH, Div Biomed & Behav Sci, Expt Toxicol Branch, 4676 Columbia Pkwy, MS-C-23, Cincinnati, Oh 45226, USA. Alterations in rabbit kidney protein expression following lead exposure as analyzed by two-dimensional gel electrophoresis. *Electrophoresis* **20:** (14) 2977.

Larsson T, Bergstrom J, Nilsson C*, Karlsson KA. 2000. *Univ Gothenburg, Inst Med Biochem, Box 440, SE-40530 Gothenburg, Sweden. Use of an affinity proteomics approach for the identification of low-abundant bacterial adhesins as applied on the Lewis$^{b}$-binding adhesin of *Helicobacter pylori*. *FEBS Lett* **469:** (2-3) 155.

Perkins DN, Pappin DJC*, Creasy DM, Cottrell JS. 1999. *ICRF, Protein Sequencing Lab, 44 Lincolns Inn Fields, London WC2A 3PX, England. Probability-based protein identification by searching sequence databases using mass spectrometry data. *Electrophoresis* **20:** (18) 3551.

Rabilloud T, Blisnick T, Heller M, Luche S, Aebersold R, Lunardi J, Braun-Breton C. 1999. CEA, Dept Biol Mol & Plant, Lab Bioenerget Cellulaire & Pathol, 17 rue Martyrs, FR-38054 Grenoble 9, France. Analysis of membrane proteins by two-dimensional electrophoresis: Comparison of the proteins extracted from normal or *Plasmodium falciparum*-infected erythrocyte ghosts. *Electrophoresis* **20:** (18) 3603.

Scheler C, Li XP, Salnikow J, Dunn MJ, Jungblut PR*. 1999. *Max Planck Inst Infect Biol, Monbijoustr 2, DE-10117 Berlin, Germany. Comparison of two-dimensional electrophoresis patterns of heat shock protein Hsp27 species in normal and cardiomyopathic hearts. *Electrophoresis* **20:** (18) 3623.

Sinha P, Hutter G, Kottgen E, Dietel M, Schadendorf D, Lage H. 1999. Humboldt Univ, Inst Lab Med & Pathobiochem, Fak Med, Klin Charite, Schumannstr 20-21, Berlin, Germany. Increased expression of epidermal fatty acid binding protein, cofilin, and 14-3-3-delta (stratifin) detected by two-dimensional gel electrophoresis, mass spectrometry and microsequencing of drug-resistant human adenocarcinoma of the pancreas. *Electrophoresis* **20:** (14) 2952.

Sinha P, Hutter G, Kottgen E, Dietel M, Schadendorf D, Lage H. 1999. Address as above. Search for novel proteins involved in the development of chemoresistance in colorectal cancer and fibrosarcoma cells in vitro using two-dimensional electrophoresis, mass spectrometry and microsequencing. *Electrophoresis* **20:** (14) 2961.

Stulik J, Koupilova K, Osterreicher J, Knizek J, Macela A, Bures J, Jandik P, Langr F, Dedic K, Jungblut PR. 1999. Purkyne Milit Med Acad,

Copyright © 2000 John Wiley & Sons, Ltd. 

Inst Radiobiol & Immunol, Trebesska 1575, CZ-50001 Hradec Kralove, Czech Republic. Protein abundance alterations in matched sets of macroscopically normal colon mucosa and colorectal carcinoma. *Electrophoresis* **20:** (18) 3638.

Unwin RD, Knowles MA, Selby PJ, Banks RE*. 1999. *St James' Univ Hosp, ICRF Canc Med Res Unit, Beckett St, Leeds, England. Urological malignancies and the proteomic-genomic interface. *Electrophoresis* **20:** (18) 3629.

Watarai H, Inagaki Y, Kubota N, Fuju K, Nagafune J, Yamaguchi Y, Kadoya T*. 2000. *Kirin Inst Brewery Co Ltd, Pharmaceut Res Lab, 3 Miyahara cho, Gunma 370 1295, Japan. Proteomic approach to the identification of cell membrane proteins. *Electrophoresis* **21:** (2) 460.

Witzmann FA, Bauer MD, Fieno AM, Grant RA, Keough TW, Kornguth SE, Lacey MP, Siegel FL, Sun YP, Wright LS, Young RS, Witten ML. 1999. Indiana Univ Purdue Univ, Dept Biol, 4601 Cent Ave, Columbus, In 47203, USA. Proteomic analysis of simulated occupational jet fuel exposure in the lung. *Electrophoresis* **20:** (18) 3659.

Zhang JZ. 2000. NIH/NIAID, Host Def Lab, 9000 Rockville Pike, Bethesda, Md 20892, USA. Protein-length distributions for the three domains of life. *Trends Genet* **16:** (3) 107.

### 12 Protein structural genomics

Brenner SE, Levitt M. 2000. Univ Calif, Dept Plant & Microbial Biol, 461A Koshland Hall, Berkeley, Ca 94720, USA. Expectations from structural genomics. *Protein Sci* **9:** (1) 197.

Koppensteiner WA, Lackner P, Wiederstein M, Sippl MJ*. 2000. *Salzberg Univ, Ctr Appl Mol Engn, Jakob Haringer Str 3, AU-5020 Salzburg, Austria. Characterization of novel proteins based on known protein structures. *J Mol Biol* **296:** (4) 1139.

Mallick P, Goodwill KE, Fitz-Gibbon S, Miller JH, Eisenberg D*. 2000. *UCLA, Mol Biol Inst, Los Angeles, Ca 90095, USA. Selecting protein targets for structural genomics of *Pyrobaculum aerophilum*: Validating automated fold assignment methods by using binary hypothesis testing. *Proc Natl Acad Sci U S A* **97:** (6) 2450.

### 13 Metabolomics

Schuster S, Fell DA, Dandekar T. 2000. Max Delbruck Ctr Mol Med, Dept Bioinformat, DE-13092 Berlin, Germany. A general definition of metabolic pathways useful for systematic organization and analysis of complex metabolic networks. *Nat Biotechnol* **18:** (3) 326.

### 14 Genomic approaches to development

Ezhova TA. 1999. MV Lomonosov State Univ, Dept Genet & Breeding, RU-119899 Moscow, Russia. *Arabidopsis thaliana* (L.) Heynh. as a model for studying genetic control of morphogenesis. *Russ J Genet* **35:** (11) 1312.

Guillemot F. 2000. Univ Strasbourg 1, CNRS INSERM, Inst Genet & Biol Mol & Cellulaire, BP 163, FR-67404 Illkirch Graffenstaden, France. Extracellular signals and transcriptional programs controlling neurogenesis (French, English Abstract). *M S-Med Sci* **16:** (2) 159.

### 15 Technological advances

Armour JAL, Sismani C, Patsalis PC, Cross G. 2000. Univ Nottingham, Queen's Med Ctr, Inst Genet, Nottingham NG7 2UH, England. Measurement of locus copy number by hybridisation with amplifiable probes. *Nucleic Acids Res* **28:** (2) 605.

Backhouse C, Caamano M, Oaks F, Nordman E, Carrillo A, Johnson B, Bay S*. 2000. *Perkin-Elmer Biosystems, 850 Lincoln Ctr Dr, Foster City, Ca 94404, USA. DNA sequencing in a monolithic microchannel device. *Electrophoresis* **21:** (1) 150.

Berndt P, Hobohm U, Langen H*. 1999. *F Hoffmann La Roche Co Ltd, Pharmaceut Res, Bioinformat, CH-4070 Basel, Switzerland. Reliable automatic protein identification from matrix-assisted laser desorption/ionization mass spectrometric peptide fingerprints. *Electrophoresis* **20:** (18) 3521.

Bhargava L, Shashikant CS, Carr JL, Juan H, Bentley KL, Ruddle FH. 1999. Genaissance Pharmaceut Inc, 5 Sci Pk, New Haven, Ct 06511, USA. Direct cloning of genomic DNA by recombinogenic targeting method using a yeast-bacterial shuttle vector, pClasper. *Genomics* **62:** (2) 285.

Biery MC, Stewart FJ, Stellwagen AE, Raleigh EA, Craig NL*. 2000. *Johns Hopkins Univ, Dept Mol Biol & Genet, 725 Nth Wolfe St, Baltimore, Md 21205, USA. A simple *in vitro* Tn7-based transposition system with low target site selectivity for genome and gene analysis. *Nucleic Acids Res* **28:** (5) 1067.

Chan C, Warlow RS, Chapuis PH, Newland RC, Bokey EL. 1999. Royal Nth Shore Hosp, Anat Pathol Dept, Pacific Highway, St Leonards, NSW 2065, Australia. Immobiline-based two-dimensional gel electrophoresis: An optimised protocol for resolution of human colonic mucosal proteins. *Electrophoresis* **20:** (17) 3467.

Chen YH, Chen SH*. 2000. *Natl Cheng Kung Univ, Dept Chem, Tainan 70101, Taiwan. Analysis of DNA fragments by microchip electrophoresis fabricated on poly(methyl methacrylate) substrates using a wire-imprinting method. *Electrophoresis* **21:** (1) 165.

De Santis R, Azzi A. 2000. Univ Florence, Dept Publ Hlth, via Morgagni 48, IT-50134 Florence, Italy. Use of denaturing gradient gel electrophoresis for human polyomavirus JC sequence analysis. *J Virol Methods* **85:** (1-2) 101.

Dolnik V, Liu SR, Jovanovich S. 1999. Molecular Dynamics, 928 East Arques Ave, Sunnyvale, Ca 94086, USA. Capillary electrophoresis on microchip. *Electrophoresis* **21:** (1) 41.

Dunn WC, Jacobson SC, Waters LC, Kroutchinina N, Khandurina J, Foote RS, Justice MJ, Stubbs LJ, Ramsey JM*. 2000. *Oak Ridge Natl Lab, Div Chem & Analyt Sci, POB 2008, Oak Ridge, Tn 37831, USA. PCR amplification and analysis of simple sequence length polymorphisms in mouse DNA using a single microchip device. *Anal Biochem* **277:** (1) 157.

Ekstrom S, Onnerfjord P, Nilsson J, Bengtsson M, Laurell T, Marko-Varga G*. 2000. *AstraZeneca, Preclin R&D, Cell & Mol Biol, POB 34, SE-22100 Lund, Sweden. Integrated microanalytical technology enabling rapid and automated protein identification. *Anal Chem* **72:** (2) 286.

Gay S, Binz PA, Hochstrasser DF, Appel RD*. 1999. *Swiss Inst Bioinformat, 1 rue Michel Servet, CH-1211 Geneva 4, Switzerland. Modeling peptide mass fingerprinting data using the atomic composition of peptides. *Electrophoresis* **20:** (18) 3527.

Goldmann T, Gonzalez JS*. 2000. *Univ Las Palmas, Dept Biochem Mol Biol & Physiol, ES-35016 Las Palmas, Gran Canaria, Spain. DNA-printing: Utilization of a standard inkjet printer for the transfer of nucleic acids to solid supports. *J Biochem Biophys Methods* **42:** (3) 105.

Guttman A, Lengyel T, Szoke M, Sasvari-Szekely M. 2000. Genet Biosyst Inc, 10171 Pacific Mesa Blvd, San Diego, Ca 92121, USA. Ultra-thin-layer agarose gel electrophoresis - II. Separation of DNA fragments on composite agarose-linear polymer matrices. *J Chromatogr A* **871:** (1-2) 289.

Hayward RE, De Risi JL, Alfadhli S, Kaslow DC, Brown PO, Rathod PK*. 2000. *Catholic Univ Amer, Dept Biol, Washington, DC 20064, USA. Shotgun DNA microarrays and stage-specific gene expression in *Plasmodium falciparum* malaria. *Mol Microbiol* **35:** (1) 6.

Heiskanen MA, Bittner ML, Chen YD, Khan J, Adler KE, Trent JM, Meltzer PS*. 2000. *NIH/Natl Human Genome Res Inst, Canc Genet Branch, 49 Convent Dr, Bethesda, Md 20892, USA. Detection of gene amplification by genomic hybridization to cDNA microarrays. *Cancer Res* **60:** (4) 799.

Heller C. 2000. Analyticon AG, Hermannswerder Haus 17, DE-14473 Potsdam, Germany. Influence of electric field strength and capillary dimensions on the separation of DNA. *Electrophoresis* **21:** (3) 593.

Copyright © 2000 John Wiley & Sons, Ltd. 

Heller MJ, Forster AH, Tu E. 2000. Nanogen Inc, 10398 Pacific Ctr Court, San Diego, Ca 92121, USA. Active microelectronic chip devices which utilize controlled electrophoretic fields for multiplex DNA hybridization and other genomic applications. *Electrophoresis* **21:** (1) 157.

Henegariu O, Bray-Ward P, Ward DC. 2000. Yale Univ, Dept Genet, 333 Cedar St, New Haven, Ct 06510, USA. Custom fluorescent-nucleotide synthesis as an alternative method for nucleic acid labeling. *Nat Biotechnol* **18:** (3) 345.

Hoving S, Munchbach M, Schmid H, Signor L, Lehmann A, Staudenmann W, Quadroni M, James P*. 2000. *ETH Zurich, Prot Chem Lab, Universitatsstr 16, CH-8092 Zurich, Switzerland. A method for the chemical generation of N-terminal peptide sequence tags for rapid protein identification. *Anal Chem* **72:** (5) 1006.

Huan B, Van Atta R, Cheng P, Wood ML, Zychlinsky E, Albagli D*. 2000. *NAXCOR, 4600 Bohannon Dr, Menlo Park, Ca 94025, USA. Photo-cross-linkable oligonucleotide probes for *in situ* hybridization assays. *Biotechniques* **28:** (2) 254.

Huang JC, Ge XJ, Sun M*. 2000. *Univ Hong Kong, Dept Zool, Pokfulam Rd, Hong Kong, Peoples Rep China. Modified CTAB protocol using a silica matrix for isolation of plant genomic DNA. *Biotechniques* **28:** (3) 432.

Huang PQ, Wall DB, Parus S, Lubman DM*. 2000. *Univ Michigan, Dept Chem, Ann Arbor, Mi 48109, USA. On-line capillary liquid chromatography tandem mass spectrometry on an ion trap/reflectron time-of-flight mass spectrometer using the sequence tag database search approach for peptide sequencing and protein identification. *J Am Soc Mass Spectrom* **11:** (2) 127.

Irie T, Oshida T, Hasegawa H, Matsuoka Y, Li T, Oya Y, Tanaka T, Tsujimoto G, Kambara H. 2000. Hitachi Ltd, Cent Res Lab, Biosyst Dept, 1-280 Higashi Koigakubo, Kokubunji, Tokyo 185 8601, Japan. Automated DNA fragment collection by capillary array gel electrophoresis array gel electrophoresis in search of differently expressed genes. *Electrophoresis* **21:** (2) 367.

Jackson JH, George R, Herring PA. 2000. Michigan State Univ, Theoret & Computat Biol Grp, East Lansing, Mi 48824, USA. Vectors of Shannon information from Fourier signals characterizing base periodicity in genes and genomes. *Biochem Biophys Res Commun* **268:** (2) 289.

Kristensen DB, Imamura K, Miyamoto Y, Yoshizato K*. 2000. *Hiroshima Univ, Dept Biol Sci, 1-3-1 Kamamiyama, Hiroshima 739 0046, Japan. Mass spectrometric approaches for the characterization of proteins on a hybrid quadrupole time-of-flight (Q-TOF) mass spectrometer. *Electrophoresis* **21:** (2) 430.

Laurent LC, Olsen MN, Crowley RA, Savilahti H, Brown PO*. 2000. *Stanford Univ, Howard Hughes Med Inst, Beckman Ctr, Stanford, Ca 94305, USA. Functional characterization of the human immunodeficiency virus type 1 genome by genetic footprinting. *J Virol* **74:** (6) 2760.

Lee P, Hudson TJ. 2000. McGill Univ, Ctr Genomics, 1650 Cedar Ave, Montreal, Quebec, Canada H3G 1A4. DNA chips in medicine and science (French). *M S Med Sci* **16:** (1) 43.

Li JJ, Wang C, Kelly JF, Harrison DJ, Thibault P*. 2000. *Natl Res Council Canada, Inst Biol Sci, 100 Sussex Dr, Ottawa, Ontario, Canada K1A 0R6. Rapid and sensitive separation of trace level protein digests using microfabricated devices coupled to a quadrupole-time-of-flight mass spectrometer. *Electrophoresis* **21:** (1) 198.

Liang DH, Song LG, Zhou SQ, Zaitsev VS, Chu B*. 1999. *SUNY, Dept Chem, Stony Brook, NY 11794, USA. Poly(*N*-isopropylacrylamide)-g-poly(ethyleneoxide) for high resolution and high speed separation of DNA by capillary electrophoresis. *Electrophoresis* **20:** (14) 2856.

Manabe T, Miyamoto H, Inoue K, Nakatsu M, Arai M. 1999. Ehime Univ, Dept Mat Sci, Matsuyama, Ehime 790 8577, Japan. Separation of human cerebrospinal fluid proteins by capillary isoelectric focusing in the absence of denaturing agents. *Electrophoresis* **20:** (18) 3677.

Meldrum DR, Evensen HT, Pence WH, Moody SE, Cunningham DL, Wiktor PJ. 2000. Univ Washington, Dept Elect Engn, Genomation Lab, Seattle, Wa 98195, USA. ACAPELLA-1K, a capillary-based submicroliter automated fluid handling system for genome analysis. *Genome Res* **10:** (1) 95.

Mueller O, Hahnenberger K, Dittmann M, Yee H, Dubrow R, Nagle R, Ilsley D. 1999. Hewlett Packard GmbH, DE-76337 Waldbronn, Germany. A microfluidic system for high-speed reproducible DNA sizing and quantitation. *Electrophoresis* **21:** (1) 128.

Neumann M, Herten DP, Dietrich A, Wolfrum J, Sauer M. 2000. Univ Heidelberg, Inst Phys Chem, Neuenheimer Feld 253, DE-69120 Heidelberg, Germany. Capillary array scanner for time-resolved detection and identification of fluorescently labelled DNA fragments. *J Chromatogr A* **871:** (1-2) 299.

Pesek JJ, Matyska MT, Swedberg S, Udivar S. 1999. San Jose State Univ, Dept Chem, San Jose, Ca 95192, USA. Protein and peptide separations on high surface area capillaries. *Electrophoresis* **20:** (12) 2343.

Pinto DM, Ning YB, Figeys D*. 2000. *Natl Res Council Canada, 1411 Oxford St, Halifax, Nova Scotia B3H 3Z1, Canada. An enhanced microfluidic chip coupled to an electrospray Qstar mass spectrometer for protein identification. *Electrophoresis* **21:** (1) 181.

Pirrung MC, Connors RV, Odenbaugh AL, Montague-Smith MP, Walcott NG, Tollett JJ. 2000. Duke Univ, Dept Chem, Levine Sci Res Ctr, Durham, NC 27708, USA. The arrayed primer extension method for DNA microchip analysis. Molecular computation of satisfaction problems. *J Am Chem Soc* **122:** (9) 1873.

Ren H, Karger AE, Oaks F, Menchen S, Slater GW*, Drouin G. 1999. *Univ Ottawa, Dept Phys, 150 Louis Pasteur, Ottawa, Ontario, Canada K1N 6N5. Separating DNA sequencing fragments without a sieving matrix. *Electrophoresis* **20:** (12) 2501.

Roda A, Guardigli M, Russo C, Pasini P, Baraldini M. 2000. Univ Bologna, Dept Pharmaceut Sci, via Belmeloro 6, IT-40126 Bologna, Italy. Protein microdeposition using a conventional ink-jet printer. *Biotechniques* **28:** (3) 492.

Rubakhin SS, Garden RW, Fuller RR, Sweedler JV*. 2000. *Univ Illinois, Dept Chem, 1209 West Calif St, Urbana, Il 61801, USA. Measuring the peptides in individual organelles with mass spectrometry. *Nat Biotechnol* **18:** (2) 172.

Ryo A, Kondoh N, Wakatsuki T, Hada A, Yamamoto N, Yamamoto M*. 2000. *Natl Def Med Coll, Dept Biochem II, 3-2 Namiki, Tokorozawa, Saitama 359 0042, Japan. A modified serial analysis of gene expression that generates longer sequence tags by nonpalindromic cohesive linker ligation. *Anal Biochem* **277:** (1) 160.

Santi E, Capone S, Mennuni C, Lahm A, Tramontano A, Luzzago A, Nicosia A*. 2000. *Ist Ric Biol Mol P Angeletti, via Pontini km 30600, IT-00040 Pomezia, Italy. Bacteriophage lambda display of complex cDNA libraries: A new approach to functional genomics. *J Mol Biol* **296:** (2) 497.

Simpson JW, Ruiz-Martinez MC*, Mulhern GT, Berka J, Latimer DR, Ball JA, Rothberg JM, Went GT. 2000. *Curagen Corp, 555 Long Wharf Dr, New Haven, Ct 06511, USA. A transmission imaging spectrograph and microfabricated channel system for DNA analysis. *Electrophoresis* **21:** (1) 135.

Stomakhin AA, Vasiliskov VA, Timofeev E, Schulga D, Cotter RJ, Mirzabekov AD*. 2000. *Argonne Natl Lab, Biochip Technol Ctr, 9700 Sth Cass Ave, Argonne, Il 60439, USA. DNA sequence analysis by hybridization with oligonucleotide microchips: MALDI mass spectrometry identification of 5mers contiguously stacked to microchip oligonucleotides. *Nucleic Acids Res* **28:** (5) 1193.

Syn CKC, Swarup S*. 2000. *Univ Singapore, Dept Biol Sci, Lower Kent Ridge Rd, SG-117600 Singapore, Rep Singapore. A scalable protocol for the isolation of large-sized genomic DNA within an hour from several bacteria. *Anal Biochem* **278:** (1) 86.

Tillett D, Burns BP, Neilan BA*. 2000. *Univ New Sth Wales, Sch Microbiol & Immunol, Sydney, NSW 2052, Australia. Optimized rapid amplification of cDNA ends (RACE) for mapping bacterial mRNA transcripts. *Biotechniques* **28:** (3) 448.

Wang XL, Sawaguchi T, Sawaguchi A. 2000. Tokyo Womens Med

Copyright © 2000 John Wiley & Sons, Ltd. 

Univ, Sch Med, Shinjuku ku, 8-1 Kawada cho, Tokyo, Japan. Application of electrophoresis technology to DNA analysis. *Electrophoresis* **21:** (2) 334.

Williams C, Addona TA. 2000. Millennium Pharmaceut, Proc Technol, 640 Memorial Dr, Cambridge, Ma 02139, USA. The integration of SPR biosensors with mass spectrometry: Possible applications for proteome analysis. *Trends Biotechnol* **18:** (2) 45.

Zhang BL, Foret F*, Karger BL. 2000. *Northeastern Univ, Barnett Inst, Boston, Ma 02115, USA. A microdevice with integrated liquid junction for facile peptide and protein analysis by capillary electrophoresis/electrospray mass spectrometry. *Anal Chem* **72:** (5) 1015.

### 16 Bioinformatics

Baldi P, Brunak S, Frasconi P, Soda G, Pollastri G. 1999. Univ Calif, Dept Informat & Comp Sci, Irvine, Ca 92697, USA. Exploiting the pase and the future in protein secondary structure prediction. *Bioinformatics* **15:** (11) 937.

Bansal AK. 1999. Kent State Univ, Dept Math & Comp Sci, Kent, Oh 44242, USA. An automated comparative analysis of 17 complete microbial genomes. *Bioinformatics* **15:** (11) 900.

Cariaso M, Folta P*, Wagner M, Kuczmarski T, Lennon G. 1999. *Lawrence Livermore National Lab, Biol & Biotechnol Prog, Livermore, Ca 94550, USA. IMAGEne I: Clustering and ranking of IMAGE, cDNA clones corresponding to known genes. *Bioinformatics* **15:** (12) 965.

Combet C, Blanchet C, Geourjon C, Deleage G. 2000. Inst Biol & Chim Prot, CNRS UPR 412, Lab Conformat Prot, 7 Passage Vercors, FR-69367 Lyon 07, France. NPS@: Network Protein Sequence Analysis. *Trends Biochem Sci* **25:** (3) 147.

Cordwell SJ, Nouwens AS, Verrills NM, McPherson JC, Hains PG, Van Dyk DD, Walsh BJ. 1999. MacQuarie Univ, Australian Proteome Anal Facil, North Ryde, NSW 2109, Australia. The microbial proteome database: An automated laboratory catalogue for monitoring protein expression in bacteria. *Electrophoresis* **20:** (18) 3580.

Corpet F, Gouzy J, Khan D. 1999. Ctr INRA Toulouse, Lab Genet Cellulaire, BP 27, FR-31326 Castanet Tolosan, France. Browsing protein families via the ‘Rich Family Description’ format. *Bioinformatics* **15:** (12) 1020.

Coward E. 1999. Univ Versailles, Lab Genome & Informat, 45 Ave Etats Unis, FR-78035 Versailles, France. Shufflers: Shuffling sequences while conserving the k-let counts. *Bioinformatics* **15:** (12) 1058.

Eriksson J, Chait BT, Fenyo D*. 2000. *Rockefeller Univ, 1230 York Ave, New York, NY 10021, USA. A statistical basis for testing the significance of mass spectrometric protein identification results. *Anal Chem* **72:** (5) 999.

Fountoulakis M, Schuller E, Hardmeier R, Berndt P, Lubec G. 1999. Hoffmann La Roche AG, Genomics Technol, CH-4070 Basel, Switzerland. Rat brain proteins: Two-dimensional protein database and variations in the expression level. *Electrophoresis* **20:** (18) 3572.

Gras R, Muller M, Gasteiger E, Gay S, Binz PA, Bienvenut W, Hoogland C, Sanchez JC, Bairoch A, Hochstrasser DF, Appel RD. 1999. Swiss Inst Bioinformat, Univ Med Ctr, 1 rue Michel Servet, CH-1211 Geneva 4, Switzerland. Improving protein identification from peptide mass fingerprinting through a parameterized multi-level scoring algorithm and an optimized peak detection. *Electrophoresis* **20:** (18) 3535.

Grunau C, Schattevoy R, Mache N, Rosenthal A*. 2000. *Inst Mol Biotechnol, Dept Genome Anal, Beutenbergstr 11, DE-07745 Jena, Germany. MethTools: A toolbox to visualize and analyze DNA methylation data. *Nucleic Acids Res* **28:** (5) 1053.

Gusev VD, Nemytikova LA, Chuzhanova NA. 1999. Russian Acad Sci, Inst Math, RU-630090 Novosibirsk, Russia. On the complexity measures of genetic sequences. *Bioinformatics* **15:** (12) 994.

Halperin E, Faigler S, Gill More R*. 1999. *Compugen Ltd, 72 Pinchas Rosen St, IL-69512 Tel Aviv, Israel. FramePlus: Aligning DNA to protein sequences. *Bioinformatics* **15:** (11) 867.

Ivens A, Aslett M, Wood V. 2000. Address not available. Functional websites for parasite genome projects. *Parasitol Today* **16:** (3) 93.

Junker VL, Apweiler R, Bairoch A. 1999. European Bioinformat Inst, EMBL Outstn, Wellcome Trust Genome Campus, Hinxton, Cambridge CB10 1SD, England. Representation of functional information in the SWISS-PROT data bank. *Bioinformatics* **15:** (12) 1066.

Ladunga I. 1999. SmithKline Beecham Pharmaceut, Bioinformat Dept, King of Prussia, Pa 19406, USA. PHYSEAN: PHYsical SEquence ANalysis for the identification of protein domains on the basis of physical and chemical properties of amino acids. *Bioinformatics* **15:** (12) 1028.

Lim A, Zhang LX. 1999. Natl Univ Singapore, Bioinformat Ctr, SG-119074 Singapore, Rep Singapore. WebPHYLIP: A web interface to PHYLIP. *Bioinformatics* **15:** (12) 1068.

Lukashin AV, Rosa JJ. 1999. Biogen Inc, 14 Cambridge Ctr, Cambridge, Ma 02142, USA. Local multiple sequence alignment using dead-end elimination. *Bioinformatics* **15:** (11) 947.

Oliver JL, Roman Raldan R, Perez J, Bernaola-Galvan P. 1999. Univ Granada, Dept Genet, ES-18071 Granada, Spain. SEGMENT: Identifying compositional domains in DNA sequences. *Bioinformatics* **15:** (12) 974.

Pavy N, Rombauts S, Dehais P, Mathe C, Ramana DVV, Leroy P, Rouze P*. 1999. *State Univ Ghent, INRA Lab Assoc, Kl Ledeganckstr 35, BE-9000 Ghent, Belgium. Evaluation of gene prediction software using a genomic data set: Application to *Arabidopsis thaliana* sequences. *Bioinformatics* **15:** (11) 887.

Pazos F, Rost B, Valencia A. 1999. CSIC, CNB, Prot Design Grp, ES-28049 Madrid, Spain. A platform for integrating threading results with protein family analyses. *Bioinformatics* **15:** (12) 1062.

Pearl F, Todd AE, Bray JE, Martin ACR, Salamov AA, Suwa M, Swindells MB, Thornton JM, Orengo CA*. 2000. *UCL, Dept Biochem & Mol Biol, Gower St, London WC1E 6BT, England. Using the CATH domain database to assign structures and functions to the genome sequences. *Biochem Soc Trans* **28:** (2) 269.

Peshkin L, Gelfand MS. 1999. Brown Univ, Providence, RI 02912, USA. Segmentation of yeast DNA using hidden Markov models. *Bioinformatics* **15:** (12) 980.

Sanchez R, Sali A. 1999. Rockefeller Univ, Lab Mol Biophys, 1230 York Ave, New York, NY 10021, USA. MODBASE: A database of comparative protein structure models. *Bioinformatics* **15:** (12) 1060.

Schaffer AA, Wolf YI, Ponting CP, Koonin EV, Aravind L, Altschul SF. 1999. NIH/Natl Lib Med, Natl Ctr Biotechnol Informat, Bethesda, Md 20894, USA. IMPALA: Matching a protein sequence against a collection of PSI-BLAST-constructed position-specific score matrices. *Bioinformatics* **15:** (12) 1000.

Shmatkov AM, Melikyan AA, Chernousko FL, Borodovsky M*. 1999. *Georgia Inst Technol, Sch Biol, Atlanta, Ga 30033, USA. Finding prokaryotic genes by the ‘frame-by-frame’ algorithm: Targeting gene starts and overlapping genes. *Bioinformatics* **15:** (11) 874.

Sutcliffe JG, Foye PE, Erlander MG, Hilbush BS, Bodzin LJ, Durham JT, Hasel KW. 2000. Scripps Clin & Res Inst, Dept Mol Biol, 10550 Nth Torrey Pines Rd, La Jolla, Ca 92037, USA. TOGA: An automated parsing technology for analyzing expression of nearly all genes. *Proc Natl Acad Sci U S A* **97:** (5) 1976.

Thanaraj TA. 2000. *European Bioinformat Inst, Wellcome Trust Genome Campus, Cambridge CB10 1SD, England. Positional characterisation of false positives from computational prediction of human splice sites. *Nucleic Acids Res* **28:** (3) 744.

Van Helden J, Abdre B, Collado-Vides J. 2000. Univ Libre Bruxelles, Unite Conform Macromolec Biol, CP160/16, 50 Ave FD Roosevelt, BE-1050 Bruxelles, Belgium. A web site of the computational analysis of yeast regulatory sequences. *Yeast* **16:** (2) 177.

Yada T, Nakao M, Totoki Y, Nakai K*. 1999. *Univ Tokyo, Inst Med Sci, Genome Sci Ctr, Minato ku, Tokyo 108 8639, Japan. Modeling and predicting transcriptional units of *Escherichia coli* gene using hidden Markov models. *Bioinformatics* **15:** (12) 987

Copyright © 2000 John Wiley & Sons, Ltd.
